# Necrotizing fasciitis of the neck and emphysematous osteomyelitis of sternoclavicular joint: a rare case and peculiar imaging findings

**DOI:** 10.1093/bjrcr/uaag001

**Published:** 2026-01-19

**Authors:** Marwah Algodi, Anas Al Zubaidi, Ida Azizkhanian, Huda Al Jadiry

**Affiliations:** Department of Medicine, Jersey Shore University Medical Center, NJ, United States; Department of Radiology, University of Texas Medical Branch, TX, United States; Department of Radiology, University of Texas Medical Branch, TX, United States; Department of Radiology, University of Texas Medical Branch, TX, United States

**Keywords:** necrotizing fasciitis, sternoclavicular joint, emphysematous osteomyelitis, sepsis, radiology, CT imaging

## Abstract

Emphysematous osteomyelitis accompanied by necrotizing fasciitis (NF) is a rare life-threatening condition requiring immediate recognition and aggressive management. We report a case of a 47-year-old male who initially presented to the Emergency Department (ED) with fever, shoulder pain, and leukocytosis. The patient was discharged with analgesics for presumed musculoskeletal pain after an unremarkable chest radiograph. Over the following days, he developed a progressive chest and neck erythema, prompting his return to the ED. Bloodwork revealed a progression of leukocytosis. Contrast-enhanced CT identified emphysematous osteomyelitis of the first rib with sternoclavicular joint infection, accompanied by extensive NF of the upper mediastinum and lower neck, necessitating multiple emergent surgical interventions. This case underscores the importance of early cross-sectional imaging in suspected osseous and deep soft tissue infections, as plain radiography may appear deceptively normal. It also highlights the radiology’s pivotal role in assessing disease severity and guiding timely, potentially life-saving intervention.

## Case report

A 47-year-old male with uncontrolled type 2 diabetes mellitus (A1C 11.5), hypertension, congestive heart failure, and morbid obesity presented to the emergency department with severe right shoulder and neck pain limiting deep respirations. Laboratory results showed severe leukocytosis (WBC 19.9 × 10^9^/L) and hyperglycemia (glucose 219 mg/dL), with negative COVID-19 and flu tests. He received conservative management for presumed musculoskeletal pain and was discharged home after a chest radiograph was found unremarkable. Five days later, he returned with persistent shoulder pain, fever, odynophagia, dysphagia, and rapidly progressing erythema of the right upper chest wall and lower neck associated with worsening leukocytosis (WBC 26.47 × 10^9^/L) and hyperglycemia (glucose 529 mg/dL).

**Figure 1.**
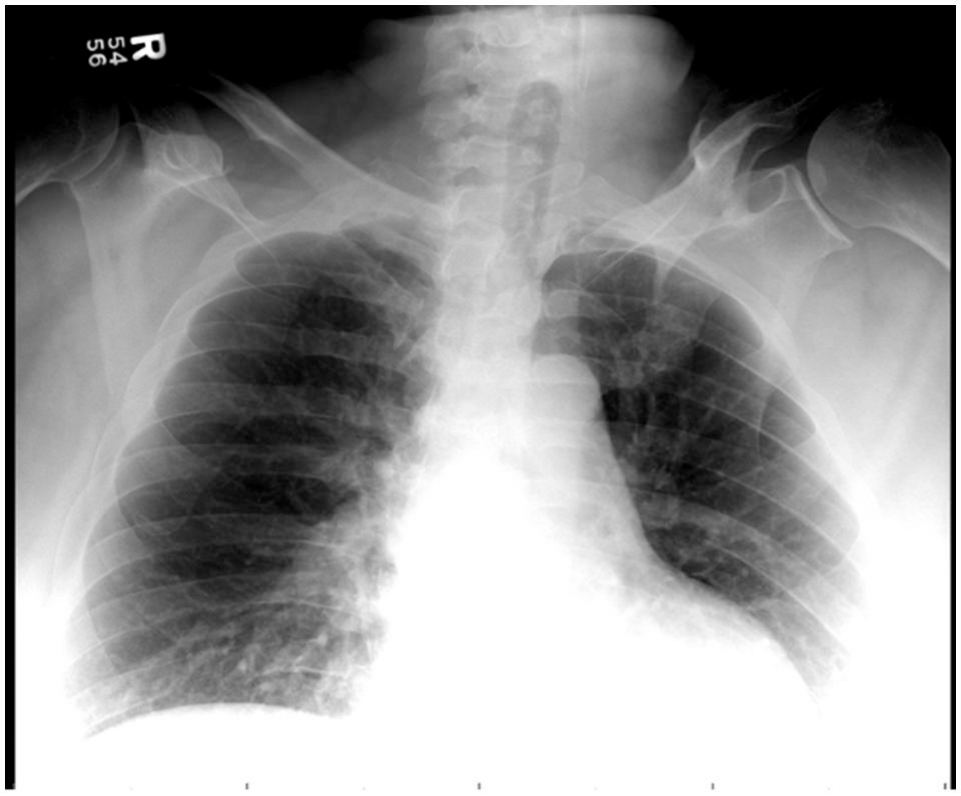
Radiograph of the chest obtained during the first few days of symptom onset appears normal. However, a normal radiograph cannot exclude potentially life-threatening pathology. Additional imaging should be obtained when there is a clinical concern for osteomyelitis and/or necrotizing fasciitis.

**Figure 2.**
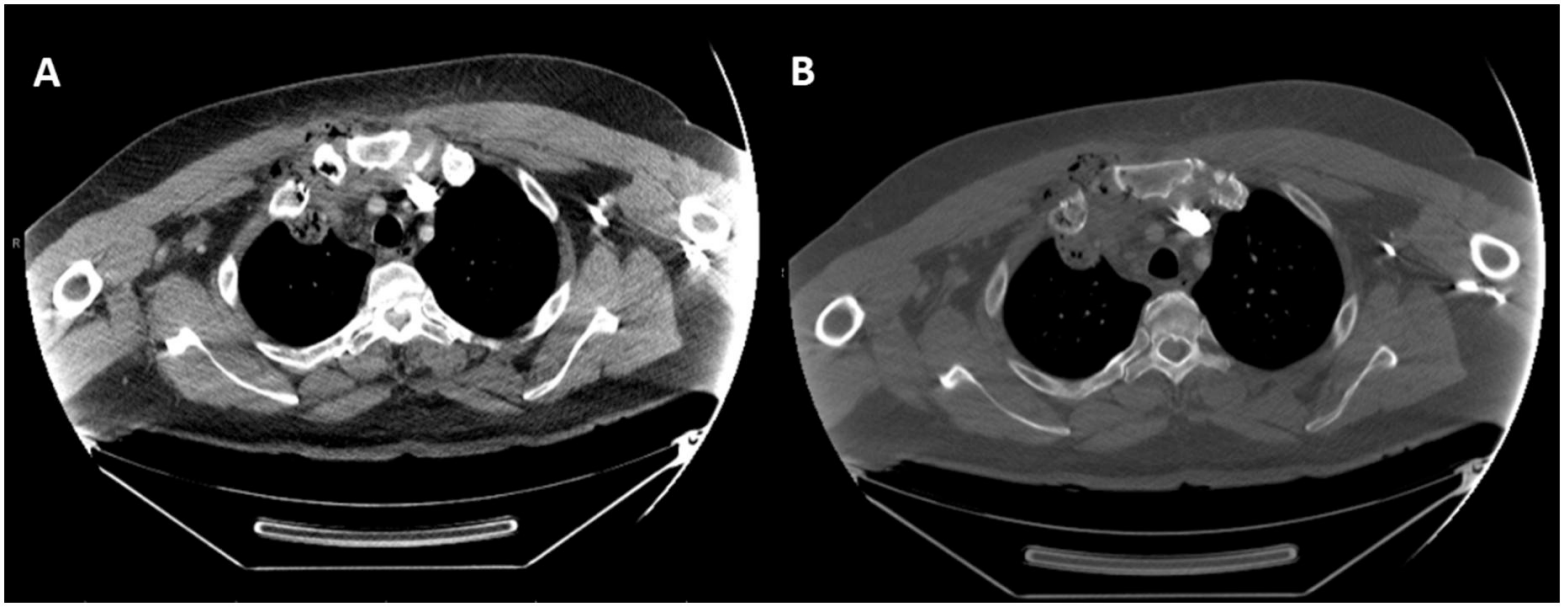
Contrast-enhanced CT of the chest in soft tissue (A) and bone (B) windows demonstrating intraosseous gas and cortical erosions of the right first rib, right clavicular head, and adjacent manubrium, highly concerning for emphysematous osteomyelitis. Additionally, emphysematous septic arthritis of the right sternoclavicular joint is evident by swelling of the joint and the intraarticular gas. Associated with stranding and emphysema affecting the surrounding soft tissues, the right pectoralis major, and the anterior upper mediastinum, concerning for necrotizing fasciitis.

**Figure 3.**
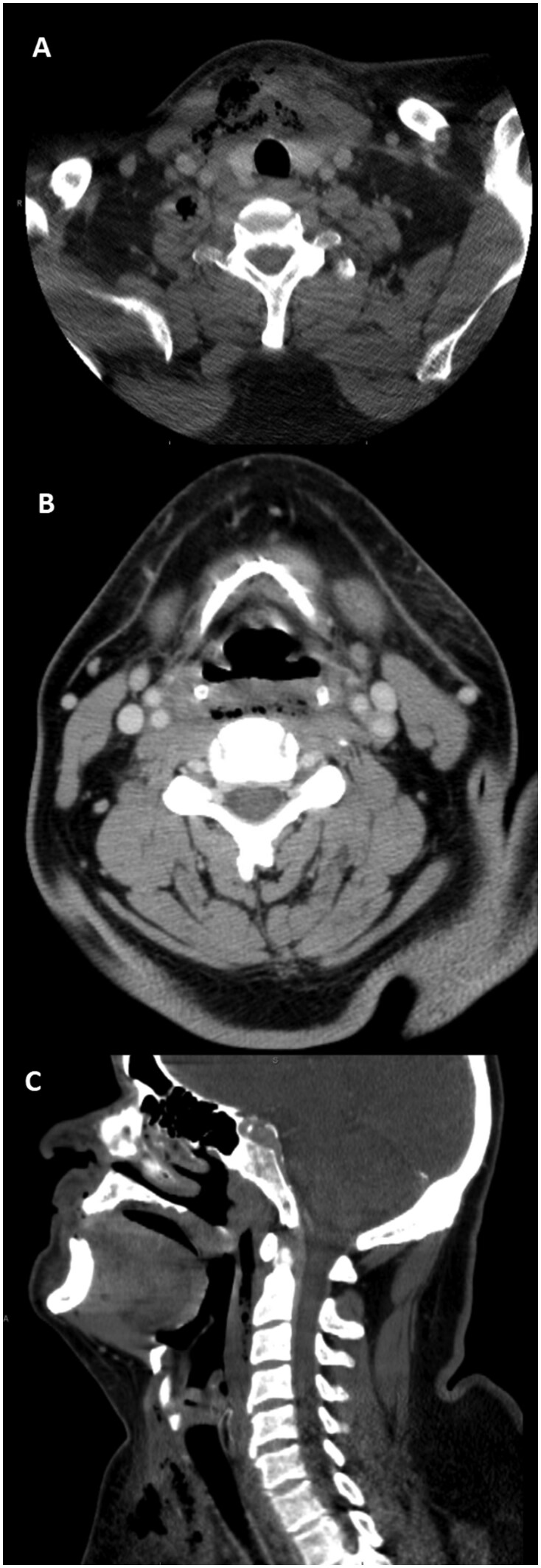
Contrast-enhanced axial (A and B) and coronal (C) CT of the neck demonstrate emphysema of the deep soft tissue of the anterior lower neck involving the strap muscles and extending to the right supraclavicular and infraclavicular region with fat stranding and retropharyngeal emphysematous effusion. Highly concerning for extensive, necrotizing fasciitis.

The initial chest radiograph appeared unremarkable (Figure 1). Contrast-enhanced CT of the neck and chest revealed intraosseous emphysema and cortical erosions of the right first rib, clavicle, and sternum, with sternoclavicular joint swelling and emphysematous effusion, highly concerning for emphysematous osteomyelitis and septic arthritis (Figure 2). Accompanied by extensive subcutaneous and myofascial emphysema of the pertinent chest wall and upper anterior mediastinum extending into the anterior lower neck and retropharyngeal space, consistent with necrotizing fasciitis (NF) (Figure 3). Broad-spectrum antibiotics were started, and emergent surgical intervention was performed by the multidisciplinary team. The diagnosis was confirmed intraoperatively by the presence of necrotic soft and osseous tissues, purulence, and abscess pockets, mirroring the distribution observed on the CT scan. Serial debridement, resection, and washout of the involved tissues and abscess drainage were performed. Cultures grew *Escherichia coli*, *Streptococcus anginosus*, and *Candida dubliniensis*. The patient remained in the ICU for continued care and was discharged in stable condition after completing IV cefazolin and micafungin.

## Discussion

Emphysematous osteomyelitis (EO) is a rare, life threatening form of osteomyelitis caused by gas-forming organisms with Enterobacteriaceae, particularly *E. coli*, or anaerobes being the primary culprits.^1^ EO alone carries a mortality rate of 43.1%, while NF has been associated with mortality rates around 12.6%.^1^^,^^2^ The combination of these 2 aggressive infections, as seen in our case, likely represents an even higher risk scenario, though specific mortality data for this combined presentation is limited due to its rarity.

The diagnosis of OM requires a high index of suspicion, given its nonspecific presentation. New or worsening local pain in febrile patients with elevated inflammatory markers or bacteremia should prompt clinical diagnosis, particularly when comorbidities are present, such as diabetes.^3^^,^^4^ Both EO and NF show a strong association with diabetes mellitus and uncontrolled hyperglycemia. Diabetes is reported in 40-70% of EO cases and up to 60% of NF cases, reflecting impaired neutrophil function, vascular compromise, and tissue hypoxia as predisposing factors.^1–3^ Our patient’s poorly controlled diabetes (A1C 11.5%) likely contributed to the aggressive course and rapid progression of infection.

A loss of 30-50% of bone mineralization, which may take 10-14 days, is needed for OM to be noticeable on plain films, necessitating further imaging.^5^^,^^6^ Early cross-sectional imaging, particularly CT, is critical for timely diagnosis, delineating the extent of disease, identifying potentially devastating complications, and guiding management. CT remains the gold standard for detecting intraosseous gas and defining disease extent.^7–12^ In this case, plain radiography was deceptively normal, whereas contrast-enhanced CT promptly identified intraosseous gas within the first rib and sternoclavicular joint, delineated osseous destruction, and assessed fascial and mediastinal extension. These features directly informed surgical urgency and guided multidisciplinary management.

While non-contrast CT can demonstrate intraosseous gas and cortical destruction, contrast-enhanced CT (CECT) provides crucial additional information by delineating rim-enhancing abscesses, non-enhancing necrotic tissue, and fascial plane enhancement patterns.^10^^,^^11^ In this case, CECT confirmed multiple rim-enhancing collections and areas of non-enhancement along the anterior mediastinum and pectoral fascia, signifying necrosis and guiding urgent surgical debridement. Hence, CECT was essential not only for diagnosis but also for surgical planning and prognostication.

MRI is superior for early detection given its higher sensitivity for soft tissue swelling, blurring of the tissue planes, and bone marrow edema which may represent the only initial manifestation. CT remains invaluable in emergency assessment and superior in detecting small areas of osteolysis in cortical bone and intraosseous small foci of gas ancillary to the diagnosis of EO.^7–9^ Additionally, CT is the mainstay for diagnosing NF. The lack of enhancement of the involved fascia and gas within fluid collections along fascial planes in the absence of trauma and iatrogenic causes are the most imaging-specific findings, though not always present. Asymmetric fascial thickening and fat stranding are more commonly observed but nonspecific.^10–13^ Surgical confirmation by unopposed manual dissection of deep fascial planes and identifying necrotic tissues during surgery confirms the diagnosis of NF.^14^

Review of the literature indicates that fewer than 50 cases of emphysematous osteomyelitis have been reported to date, with only isolated reports describing concurrent NF of the cervicothoracic region. The majority involve diabetic or immunocompromised patients and most commonly affect the spine, pelvis, and long bones rather than the sternoclavicular joint. This case, therefore, represents an exceptionally rare anatomic presentation and reinforces the importance of prompt CT evaluation to prevent mediastinal and cervical extension.^1^^,^^7–9^^,^^11–13^

This case emphasizes the need for high clinical suspicion in high-risk patients and highlights the unique diagnostic and prognostic role of CT scan in rapidly identifying emphysematous infection and directing life-saving surgical care.

### Differential diagnoses

Differential diagnostic considerations include traumatic gas introduction and post-procedural changes. However, the absence of trauma or recent procedures, combined with the characteristic distribution of gas within bone and along fascial planes, strongly favored the diagnosis of emphysematous osteomyelitis with NF. When gas is absent, the differential broadens to include cellulitis when thickening and edema are confined to the subcutaneous tissue, and non-NF when the deep fascia is involved with preserved enhancement.^11^^,^^12^

## Learning point

- From a practical standpoint, our case underscores several key radiological principles in the diagnosis of emphysematous osteomyelitis.
- First, diagnosis is primarily clinical, yet it can be challenging. A high index of suspicion must be maintained.
- Second, CT imaging should be obtained promptly when emphysematous infection is suspected, as it remains the gold standard for detecting and characterizing EO and NF and evaluating potential extension into adjacent compartments, particularly the mediastinum and neck, where rapid spread can lead to life-threatening complications.
